# Potential Roles and Key Mechanisms of Hawthorn Extract against Various Liver Diseases

**DOI:** 10.3390/nu14040867

**Published:** 2022-02-18

**Authors:** Eujin Kim, Eungyeong Jang, Jang-Hoon Lee

**Affiliations:** 1 Department of Clinical Korean Medicine, Graduate School, Kyung Hee University, 26 Kyungheedae-ro, Dongdaemun-gu, Seoul 02447, Korea; djwls409@khu.ac.kr; 2 Department of Internal Medicine, Kyung Hee University Korean Medicine Hospital, 23 Kyungheedae-ro, Dongdaemun-gu, Seoul 02447, Korea; obliviona79@naver.com; 3 Department of Internal Medicine, College of Korean Medicine, Kyung Hee University, 26 Kyungheedae-ro, Dongdaemun-gu, Seoul 02447, Korea

**Keywords:** hawthorn, *Crataegus*, liver, antioxidant

## Abstract

The genus *Crataegus* (hawthorn), a flowering shrub or tree, is a member of the Rosaceae family and consists of approximately 280 species that have been primarily cultivated in East Asia, North America, and Europe. Consumption of hawthorn preparations has been chiefly associated with pharmacological benefits for cardiovascular diseases, including congestive heart failure and angina pectoris. Treatment with hawthorn extracts can be related to improvements in the complex pathogenesis of various hepatic and cardiovascular disorders. In this regard, the present review described that the presence of hawthorn extracts ameliorated hepatic injury, lipid accumulation, inflammation, fibrosis, and cancer in an abundance of experimental models. Hawthorn extracts might have these promising activities, largely by enhancing the hepatic antioxidant system. In addition, several mechanisms, including AMP-activated protein kinase (AMPK) signaling and apoptosis, are responsible for the role of hawthorn extracts in repairing the dysfunction of injured hepatocytes. Specifically, hawthorn possesses a wide range of biological actions relevant to the treatment of toxic hepatitis, alcoholic liver disease, non-alcoholic fatty liver disease, and hepatocellular carcinoma. Accordingly, hawthorn extracts can be developed as a major source of therapeutic agents for liver diseases.

## 1. Introduction

Liver disease is associated with high prevalence, increased mortality, and a substantial health care burden. By 2017, approximately 1.5 billion people were suffering from chronic liver disease (CLD), which, along with liver cirrhosis, accounts for 2 million annual deaths worldwide [1,2]. Non-alcoholic fatty liver disease (NAFLD) accounts for a major component (60%) of CLD, other components of which include hepatitis B and C viruses (38%) and alcohol consumption (2%) [1]. Acute liver failure is mainly a consequence of viral infection and drug-induced liver injury in the developing and developed worlds, respectively [3]. Although vaccination and novel antiviral agents have lowered the incidence of virus-induced liver diseases, including cirrhosis, considerable risk factors, including metabolic syndrome, obesity, and alcohol and drug misuse/overuse, still lead to various liver disorders [4,5]. However, there are no approved drugs currently available for managing various liver diseases, including NAFLD, non-alcoholic steatohepatitis (NASH), alcoholic liver disease (ALD), liver cirrhosis, and hepatocellular carcinoma (HCC).

A wide spectrum of risk factors affecting the liver, including obesity, drugs, alcohol, environmental pollutants, irradiation, and toxicants, can induce excessive oxidative stress in liver tissues and perturb the hepatic defense system against oxidative damage [6]. In this context, multiple antioxidants, such as vitamin E, zinc, selenium, methionine, coenzyme Q10, silymarin, and *N*-acetylcysteine, have been hypothesized and used as main drugs or dietary supplements in treating liver diseases [6]. Specifically, most antioxidants possess remarkable hepatoprotective, antisteatotic, anti-inflammatory, and antifibrotic effects, as well as striking properties against oxidative damage. Hence, oxidative stress can be a key component of the molecular pathogenesis of liver damage, and altering the imbalance of production and removal of hepatic free radicals might be useful in preventing and treating various liver diseases. In addition, a variety of signaling pathways, including hepatic cholesterol maintenance, AMP-activated protein kinase (AMPK), and apoptosis, have been implicated in the pathogenesis of liver diseases [7,8,9].

Currently, approximately 50% of therapeutic drugs against liver diseases originate from natural plants [10]. This high predominance may be correlated with the abundance of multiple constituents with excellent pharmacological properties, including antioxidant action. Hawthorn (a common name indicating *Crataegus* species) is a promising medicinal plant, displaying substantial efficacy in interfering with the pathogenesis of liver diseases. In fact, although hawthorn has long been used as an alternative medicine to treat abdominal pain, dyspepsia, angina, and congestive heart failure, it also reportedly participates in NAFLD resolution [11,12]. Recently, available preclinical evidence investigating the counter-regulation and underlying signaling pathways of hawthorn species (*C. pinnatifida* in Korea and China; *C. cuneata* in Japan; and *C. monogyna*, *C. oxyacantha*, and *C. aronia* in Europe, the US, and the Middle East) is increasing [13].

Despite the accumulating data on the efficacy of hawthorn extract and its constituents in various liver diseases, a comprehensive review on the efficacy, mechanism, and safety for clinical use of the plant for liver disease treatment has not yet been performed. Thus, the present study summarized the preclinical evidence supporting the improvement of hawthorn extract against multiple pathologies of liver disease and demonstrated related mechanisms to further understand the role of hawthorn in this type of disorder.

## 2. Phytochemistry of Hawthorn

Hawthorn contains a variety of flavonoids, triterpenoids, monoterpenoids, lignans, and organic acids. To date, more than 150 chemical compounds have been identified from its leaves, flowers, and berries [14]. The Korean pharmacopoeia and pharmacopoeia of the People’s Republic of China allow *C. pinnatifida* to be the representative species of hawthorn and ursolic acid is used for the identification test of the fruits of *C. pinnatifida* [15,16]. According to the pharmacopoeia of the People’s Republic of China, *C. pinnatifida* fruits and leaves should contain more than 5.0% of citric acid, 7.0% of flavonoids, and 0.050% of hyperoside. In Europe and the US, *C. oxycantha* and *C. monogyna* have mainly been used, and their fruits, leaves, and flowers must contain more than 1.0–1.5% flavonoids [17,18]. While South Korea, China, Europe, and the US emphasize the content of flavonoids in the genus *Crataegus*, which includes *C.* *pinnatifida, C. oxycantha*, and *C. monogyna*, hyperoside has been the most frequently identified flavonoid. Other flavonoids, such as vitexin, rutin, and ferulic acid, have been recognized as common constituents of the above hawthorn extracts [19]. In addition, remarkable quantities of triterpenoids, including ursolic acid and corosolic acid, have been quantified in Chinese and European hawthorns [20,21]. Remarkably, the analysis of the quantification and profile of chemical compounds in hawthorn plants has reported considerable divergence between various medicinal parts, including fruits, leaves, and flowers of different *C*. species. A number of individual phenolics, including rutin, hyperoside, chlorogenic acid, vitexin, and isoquercetin, using high-performance liquid chromatography (HPLC) analysis, were selected for quality assessment of more than 50 samples (leaves and flowers) of different species of hawthorn plant [22]. Similarly, 11 available compounds, including hyperoside and chlorogenic acid, were separated and quantified using HPLC-electrospray ionization mass spectrometry in hawthorn fruits [23]. However, there is still an unmet need for sufficient data on the contents and profile of major constituents of all species of hawthorn owing to both lack of accurate quality measures and insufficient sources of active compounds. Thus, further investigations need to be performed for data of chemical profiling, reference compounds, and reliable validation for quality assurance of hawthorn.

## 3. Pharmacological Properties of Hawthorn

### 3.1. Hepatoprotective Effect

The liver is an important solid organ that filters blood input from the gastrointestinal tract, decomposing toxicants and disposing of harmful materials from the body [24]. Lack of proper hepatic function due to oversupply of food, alcohol, medications, toxicants, and other harmful factors can lead to some degree of liver damage, suggesting abnormalities in the synthesis, processing, and secretory functions of hepatocytes. Hepatic injury is characterized by abnormal liver function due to increased activity of serum biochemical markers, and histological findings of damaged hepatocellular structures [25]. Reactive oxygen species (ROS) may be a key determinant in the development and aggravation of liver dysfunction because oxidative stress can primarily oxidize hepatocellular structure components, including DNA, proteins, and lipids [26]. In addition, pathological apoptosis excessively eliminates hepatocytes through the release of harmful cytokines and maximal immune responses [27].

Hawthorn extracts prevented liver damage induced by a high-fat/cholesterol/triglyceride/fructose diets, alcohol treatment, LPS, CCl_4_, cadmium, and partial hepatectomy in rodents and HepG2 cells, via inhibition of oxidative injury and apoptosis. Serum transaminase (AST and ALT) levels are frequently increased in liver injury because they leak into peripheral blood in cases of abnormal hepatocellular integrity [27]. Hence, regulating both serum markers within normal limits can represent liver protection and maintenance of liver function. The extracts of *C. oxyacantha* [28], *C. aronia* [29], *C. monogyna* [30], and *C. pinnatifida* [31,32,33,34,35] significantly decreased serum AST and ALT levels, which were markedly elevated by high-fat, high-cholesterol, high-triglyceride, and high-fructose feeding. In addition, these hawthorn species lowered the serum levels of ALP, GGT, total bilirubin, and direct bilirubin in the above animals [28,30,32,34,35]. Histological analysis of liver tissues using high fat/cholesterol/fructose diet-induced rodents revealed that hepatocytes highly express pyknotic nuclei, vacuolation, sinusoidal distension, necrosis, damaged endoplasmic reticuli, swollen and pleomorphic mitochondria, distorted intercellular spaces, irregular nuclear membranes, nuclear chromatin condensation, and cellular degeneration, suggesting that these diets contribute to liver damage. Hawthorn extracts improved these hepatic tissue injuries through antioxidant mechanisms, including the regulation of Nrf2 and ARE expression [30,36,37]. Remarkably, polyphenols obtained from hawthorn peels and flesh exerted a potent hepatoprotective action by efficiently ameliorating biochemical markers, hepatic lipid peroxidation, and liver tissue injury via the regulation of apoptosis-related proteins and antioxidant enzymes dysregulated by a high-fructose diets [38].

*C. pinnatifida* and *C. oxyacantha* are involved in the protection of liver injury after alcohol exposure [10,31,39,40,41,42]. In HepG2 cells and Sprague Dawley rats, alcohol toxicity caused increased DNA damage in liver cells, and abnormal alcohol-metabolizing enzyme activity, biochemical marker levels, and histological findings. After co-treatment of these rats with an ethanol extract of *C. pinnatifida*, ALDH activity increased in the hepatic tissues [40], with decreased levels of serum AST, ALT, and GGT [31,42], and improved cell necrosis and sinusoidal distension [31]. In addition, the extract prevented alcoholic damage to HepG2 cells through the antioxidant defense system [10] and suppressed the catalytic activity of CYP2E1 [41], despite alcohol being a strong inducer of oxidative stress and CYP enzymes. Although the *C. oxyacantha* extract exhibited similarities against alcohol inducers, the hepatoprotective effects of the extract can be distinguished by the increase in liver glycogen levels, lack of which easily causes hepatic steatosis and insulin resistance [42]. In contrast to other causative factors, including lipopolysaccharides (LPSs), CCl_4_, cadmium, and hepatectomy, which are harmful to the liver, hawthorn extracts also reversed abnormal serum markers and impaired liver tissue via the inhibition of PARP cleavage and TUNEL-positive hepatocytes, and activation of the hepatic antioxidant system [43,44,45,46,47].

**Figure 1:**
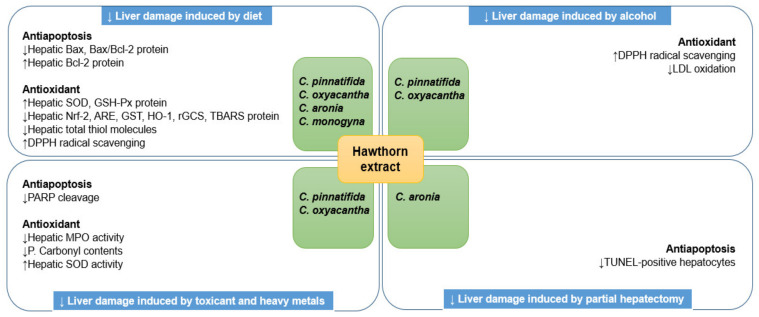
Hepatoprotective roles of hawthorn extract against liver damage induced by diet, alcohol, toxicant, heavy metals, and partial hepatectomy. ARE, antioxidant response element; Bax, Bcl-2-associated X protein; Bcl-2, B-cell lymphoma-2; DPPH, 2,2-diphenyl-1-picrylhydrazyl; GSH-Px, glutathione peroxidase; GST, glutathione S-transferase; HO-1, heme oxygenase-1; LDL, low-density lipoprotein; MPO, myeloperoxidase; Nrf-2, nuclear factor erythroid-2-related factor 2; PARP, poly-ADP ribose polymerase; P. Carbonyl, protein carbonyl; rGCS, r-glutamylcysteine synthethase; SOD, superoxide dismutase; TBARS, thiobarbituric acid reactive substances; and TUNEL, terminal deoxynucleotidyl transferase dUTP nick end labelling.

Collectively, hawthorn extracts have hepatoprotective roles in the context of hepatic injury, probably due to the underlying anti-apoptosis and antioxidant defense mechanisms of the extracts (Figure 1, Table 1). This suggests that hawthorn extracts may maintain liver function and minimize the abrogation of hepatic damage caused by various factors.

### 3.2. Antisteatotic Effect

Hepatocytes frequently become steatotic from several triggers, such as alcohol and metabolic or toxic stress. Steatosis is considered the most common and earliest stage of liver injury and is characterized by the accumulation of extra lipid droplets in the liver [48]. The presence of significant fatty infiltration in hepatic tissue is predominantly implicated in dysregulated lipid homeostasis, which is attributed to an imbalance between de novo lipogenesis and fatty acid oxidation [49]. Oxidative stress also possibly plays a pivotal role as an influential trigger for the initiation of hepatic steatosis and progression to inflammation or fibrosis [50]. Specifically, in NAFLD, the most common liver disease representing hepatic steatosis, oxidative stress, and impaired lipid metabolism may be two crucial mechanisms underlying the development of NAFLD in the progeny of obese mice [51]. Regarding these key triggers for the onset of hepatic steatosis, various transcriptional factors and hepatic enzymes have been implicated to regulate hepatic steatosis [52].

**Figure 2:**
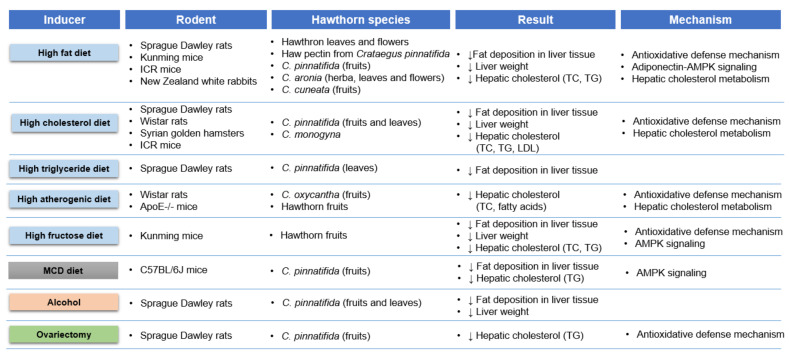
Inducer, rodent, hawthorn species, experimental results, and underlying mechanism of preclinical studies on hawthorn extract exhibiting antisteatotic activities related to liver pathogenesis. ApoE, apolipoprotein E; AMPK, AMP-activated protein kinase; LDL, low-density lipoprotein; MCD, methionine choline deficient; TC, total cholesterol; and TG, triglyceride.

Hepatic steatosis is primarily characterized by histological findings of fatty infiltration and liver weight gain. As depicted in Figure 2, hawthorn extracts significantly reduced fat and cholesterol amounts in liver tissue, thereby decreasing the liver weight in rodents exposed to diet, ovariectomy, and alcohol-induced steatosis [28,29,31,32,33,34,35,36,38,39,53,54,55,56,57,58,59,60,61,62,63,64,65,66,67,68,69,70,71,72,73]. In particular, the fruits and leaves of *C. pinnatifida* have been frequently administered for their antisteatotic effects [31,32,33,34,35,57,58,59,60,61,62,63,64,65,66,72,73]. Studies in animal models induced by excess fat intake, MCD diet, alcohol consumption, and ovariectomy have demonstrated that the species extract reduced buildup of lipid droplets and liver weight via AMPK signaling, hepatic cholesterol homeostasis, and antioxidative defense mechanisms [31,32,33,34,35,57,58,59,60,61,62,63,64,65,66,72,73]. Specifically, haw pectin isolated from *C. pinnatifida* fruits markedly suppressed excessive lipid accumulation and weight gain induced by a high-fat diet, all of which are involved in the mechanisms underlying diet-associated hepatic steatosis. First, haw pectin improved hepatic steatosis by ameliorating hepatic oxidative stress injury by increasing the tissue reserves of superoxide dismutase (SOD), catalase (CAT), glutathione (GSH), and total antioxidant capacity (TAC), which had been depleted by a high-fat diet in Kunming mice [74,75]. Second, haw pectin improved hepatic cholesterol catabolism to bile acids by increasing the levels of cholesterol 7α-hydroxylase (CYP7A1) mRNA and protein, thereby increasing hepatic bile acid levels in the gallbladder and feces [54,55,56]. In addition, haw pectin improved hepatic lipid metabolism by regulating the activity of lipogenesis-related enzymes (hepatic HMG-CoA reductase, Acyl-CoA: cholesterol acyltransferase (ACAT)), scavenger receptor class B type I (SR-BI, the HDL receptor), and hepatic ABC transporter A1 (ABCA1) expression, which promote lipid release [54,55]. Third, 5AMP-activated protein kinase (AMPK) signaling, a highly attractive target for glucolipid homeostasis to reduce hepatic steatosis, is involved in the benefits of haw pectin against fat overload in liver tissue, and PPARα, SIRT1, and NFκB may be closely associated with AMPK-dependent regulation of haw pectin in high-fat-diet-induced hepatic steatosis [34,53,76,77] (Figure 2, Table 2).

*C. aronia*, *C. cuneata*, and *C. oxyacantha* extracts also reduced the susceptibility of hepatocytes to steatosis induced by high-fat and high-atherogenic diets, mainly via mediating hepatic activity of HMG-CoA reductase, ACAT, and CYP7A1; hepatic cholesterol biosynthesis; and lipid excretion into bile acids [30,37,68,69,70]. *C. aronia* and *C. monogyna* attenuated hepatic fat content by regulating hepatic GSH and TBARS and hepatic total thiol molecules, respectively, in Wistar rats [30,37]. Specifically, the antioxidant activity of 200 mg/kg *C. aronia* was more potent than that of 4 mg/kg of simvastatin [37] (Figure 2, Table 2).

Oxidative stress and hepatic cholesterol metabolism dysfunction often cause fatty livers. Hawthorn reduces fat deposition in liver tissue by altering multiple intrahepatic factors associated with ROS generation, antioxidant defenses, lipogenesis, fatty acid oxidation, fatty acid uptake, and bile acid efflux. This review suggests that haw pectin obtained from *Crataegus pinnatifida* fruits may be effective in decreasing hepatic fat storage. Further animal and human studies investigating the additional efficacy and various genes that encode markers leading to hepatic steatosis are required. In addition, further studies regarding the role of *C. pinnatifida* on the interactions between the gut and liver in the management of hepatic steatosis are needed as the extract decreases hepatic total cholesterol levels by impeding cholesterol absorption from the gut through the suppression of fibroblast growth factor receptor 4 (FGFR4) mRNA and protein.

### 3.3. Anti-Inflammatory and Antifibrotic Effects

Hepatic injury and steatosis are commonly accompanied by inflammation and fibrosis. While hepatic inflammatory and fibrotic activation are essential for tissue repair and wound healing in response to injury, dysregulation and overexpression of inflammatory and fibrotic mediators in liver tissue can play a crucial role in accelerating liver damage [78]. Hepatic inflammation can be induced by oxidative stress, apoptosis, and inflammation-related signaling activation [79,80]. Initiation of inflammation is closely associated with macrophage infiltration and neutrophil recruitment to the liver, which elicit the secretion of various inflammatory cytokines. Overproduction of pro-inflammatory cytokines and chemokines in the liver generates and enhances hepatic fibrosis, mediated by active hepatic stellate cells (HSCs) and multiple extracellular matrix (ECM) deposition [81]. Hence, alteration in liver inflammation is regarded as an undoubtedly important target for improving liver disorders and preventing fibrogenic reactions.

**Figure 3:**
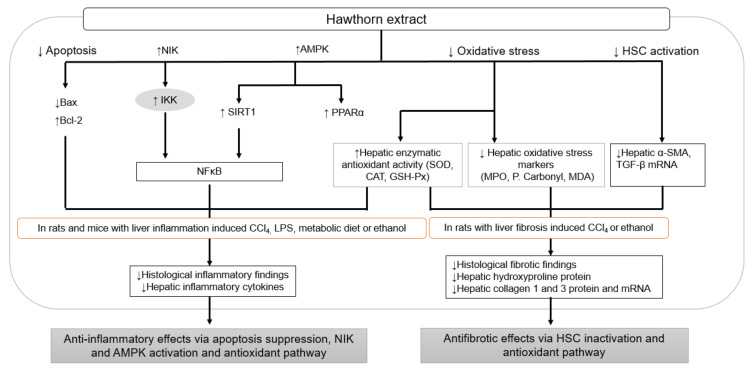
Anti-inflammatory effects and antifibrotic effects of hawthorn extract through apoptosis suppression, AMPK and NIK activation, HSC inactivation, and antioxidant defense system. AMPK, AMP-activated protein kinase; Bax, Bcl-2-associated X protein; Bcl-2, B-cell lymphoma-2; CAT, catalase; CCl_4_, carbon tetrachloride; GSH-Px, glutathione peroxidase; HSC, hepatic stellate cell; IKK, IκB kinase; LPS, lipopolysaccharides; MDA, malondialdehyde; MPO, myeloperoxidase; NFκB, nuclear factor kappa B; NIK, NFκB inducing kinase; P. carbonyl, protein carbonyl; PPAR, peroxisome proliferator-activated receptor; SIRT, silent information regulator T; SOD, superoxide dismutase; SMA, smooth muscle actin; and TGF, transforming growth factor.

Apoptotic liver cells frequently coexist with recruited neutrophils, suggesting that apoptosis can contribute to the pathogenesis of the inflammatory response. Additionally, lipids produced by apoptotic hepatocytes can act as chemotactic substances for recruiting inflammatory cells [27]. Hawthorn extracts reversed the hepatic production of apoptosis-related proteins (Bax and Bcl-2) dysregulated by a high-fructose diet in male Kunming mice, leading to the mitigation of inflammation, mediated by the decreased levels of hepatic IL-1, IL-6, and TNF-α [38]. In addition to targeting apoptosis, reducing oxidative stress, and activating AMPK, signaling by polyphenols from hawthorn extracts was involved in regulating hepatic inflammation induced by a high-fructose diet [38]. Specifically, oxidative stress is predominantly mediated by hawthorn extracts in ameliorating hepatic inflammation and fibrosis induced by metabolic triggers (high cholesterol [30,33,57,77], atherogenic [71], and high-fructose diet [38]) and toxic substances (LPS [43], CCl_4_ [44], and 35% ethanol [42]). Hawthorn fruit, *C. monogyna*, *C. oxyacantha*, and *C. pinnatifida* possess antioxidant action in both hepatic oxidative stress markers (MDA, total thiol molecules) and enzymatic antioxidant activity (SOD, CAT, GSH-Px, and T-AOC), thereby contributing to their anti-inflammatory activities by improving inflammatory cell (neutrophil leukocyte, mononuclear cell) infiltration and regulating the secretion of inflammatory cytokines, including TNF-α, IL-1β, IL-6, IL-10, and MCP-1, against hepatic inflammation induced by LPS, CCl_4_, and metabolic diets [30,43,44]. The antioxidant defense mechanism of hawthorn leaves and *C. oxyacantha* leaves and flowers in rats administered with CCl_4_ and ethanol led to reduced hepatic fibrotic regions and collagen synthesis [44]. Specifically, *C. oxyacantha* leaves and flowers significantly reversed both CCl_4_-induced inflammation and fibrosis via the antioxidant defense system, NFκB inhibition, and HSC inactivation [44], suggesting that this plant can play a key role in suppressing ECM production and improving liver inflammation. Among several species of hawthorn, haw pectin and polyphenols from *C. pinnatifida* regulate AMPK/SIRT1/NFκB and NIK/IKK/NFκB signaling against high-fat-diet-induced hepatic inflammation, confirming their applicability in NASH treatment [77,82] (Figure 3, Table 3).

Inflammation and fibrosis are major components that develop and aggravate liver injury, and hawthorn extracts can have promising pharmacological activities against both responses via the regulation of oxidative stress, apoptosis, AMPK and NIK signaling, and HSC activation.

### 3.4. Anticancer Effects

To fight against malignant cancer and discover novel anticancer drugs, the pharmacological effects of suppressing the viability and proliferation of fast-growing cancerous cells are of utmost importance. Specifically, chemotherapy efficiently eliminates uncontrolled cancer cells by activating apoptotic pathways, cell cycle arrest, and inducing autophagic clearance. Indeed, various chemotherapeutic agents targeting these mechanisms are predominantly used because tumor cell survival often relies on aberrant function of the regulators of apoptosis, autophagy, and cell cycle progression [83,84]. In addition, elucidating the structure–activity relationship (SAR) of efficient candidates with anticancer activities might help determine their functional groups and assess their therapeutic potency to inhibit proliferation and mediate cell death pathways [85].

The antiproliferative property of hawthorn against liver cancer cells has been largely determined using a cell-based MTT assay in HepG2 and Hep3B cells. Among the various species of hawthorn, *C. pinnatifida*, *C. monogyna*, and *C. armena* have significantly suppressed the cell viability of human hepatoma cells, including HepG2 and Hep3B cells [20,86,87,88,89,90], while the water extract of *C. aronia* effectively increased the level of reduced intracellular antioxidant glutathione and glutathione disulfide in HepG2 cells, indicating no significant cytotoxicity against tumors. Interestingly, the 80% ethanol extract of *C. monogyna* exhibited dose-dependent cytotoxic activity toward HepG2 cells, although the extract had abundant polyphenolic compounds and zinc, possessing antioxidant properties [87]. In addition, the cytotoxic activity of *C. monogyna* extract was specific to HepG2 and showed no harmful effects in non-tumor normal human skin fibroblast BJ cells at the same concentration [87]. The selective cytotoxicity of HepG2 and protective effects on BJ cells of *C. monogyna* can be explained by the high amounts of bioactive antioxidant compounds and zinc present in its extract [87]. However, due to the abundant content of phenolic acid in 80% methanol extracts of *C. monogyna*, the extract might have displayed weaker cytotoxicity toward HepG2 cells, with IC_50_ values ranging from 88.45–318.72 μM, compared with those of the anticancer drug ellipticine (IC_50_, 1.21 μM) treatment [91].

Meanwhile, it should not be assumed that the potential anticancer activity of different hawthorn species is less effective than that of conventional chemotherapeutic drugs because of the high predominance of the antioxidant compounds found in their extracts. The stronger cytotoxicity of hydroxy-olean-12-en-28-oic acid triterpenoids obtained from the berries of *C. pinnatifida* (IC_50_ < 5 μM) was confirmed by comparing its IC_50_ with that of the positive control cisplatin (IC_50_ > 5 μM) using the MTT assay implemented in HepG2 cells [88]. In addition, lignans, a group of phenolic constituents found in the extract of the seeds of *C. pinnatifida* showed stronger growth inhibitory activities (IC_50_, 30.96–39.97 μM) than 5-fluorouracil (IC_50_ = 40.34 μM) against HepG2 cells [92,93]. Hence, the selective toxicity toward hepatoma cells and the potent effectiveness of hawthorn extract might be a therapeutic advantage compared to conventional anticancer drugs.

The cytotoxic properties of phenylpropanoids from *C. pinnatifida* fruits in HepG2 and Hep3B cells via proapoptotic signaling, autophagy induction, and cell cycle arrest are considered noteworthy [89,94]. Similarly, the ethanol extract of *C. pinnatifida* fruits exerted inhibitory activities against the viability of HepG2 cells, and the effects were supported by apoptosis induction by regulating caspase-3 activity and genetic expression of Bax and Bcl-2 [86]. Phenylpropanoids may have been partly responsible for the anticancer effects of the *C. pinnatifida* extract against HepG2 cells. Interestingly, some phytochemical results suggest a possible SAR that might help understand the pharmacological activities of phenylpropanoids in attacking hepatic cancer cells [94]. For example, the distinctive cytotoxicity of crataegusanoids A and B, which belong to phenylpropanoids, can be increased by the presence of the phenyl group at C-7″ and (*E*)-2-styryl, respectively, against two hepatoma cell lines. In addition, SAR analysis of crataegusoids C and D showed that two methoxy-substituted groups at the C-3′ position were associated with potent cytotoxic activities of both compounds against HepG2 cells [94].

**Figure 4:**
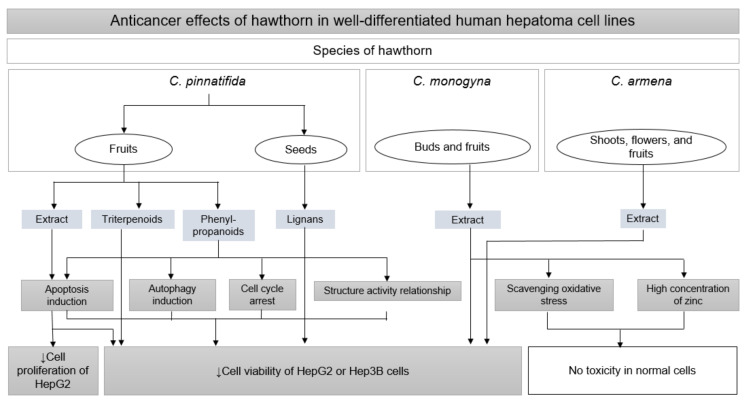
Anticancer effects of hawthorn extract by inhibiting cell viability and cell proliferation in human hepatoma cell lines.

In summary, *C. pinnatifida*, *C. monogyna*, and *C. armena* exhibited significant anticancer effects on human hepatoma cells, whose activity was comparable to that of conventional chemotherapy to treat HCC. In addition, because of the abundant amounts of antioxidant constituents found in the extract and chemical compound groups of these plants, hawthorn treatment could result in selective cytotoxicity in HepG2 cells. The antioxidant property of hawthorn suggests its relative safety, even when prescribed for a long time or co-administered with anticancer drugs. The antitumor activity of hawthorn, triterpenoids, phenylpropanoids, and lignans isolated from *C. pinnatifida* might be potent candidates contributing to the cytotoxicity of hawthorn. Among the four pentacyclic triterpenoids, the IC_50_ of corosolic acid was the lowest in HepG2 cells (Figure 4, Table 4). Thus, further in-depth investigations of the molecular mechanisms, pharmacology against drug resistance, and SAR related to anticancer activities of various materials, including corosolic acid, are required.

## 4. Safety of Hawthorn

It is necessary to evaluate and confirm the safety of hawthorn for its use as a therapeutic agent for various liver diseases. When the water extract of *C. pinnatifida* fruits (126 g/kg crude drugs) was administered to Kunming mice, none of the subjects died, and when the water extracts of *C. cuneata* and *C. scabrifolia* were administered to Kunming mice, only one subject died. The 50% lethal dose (LD_50_) of crude drugs exceeded 126 g/kg; therefore, the toxicity of hawthorn fruit is expected to be very low [95]. The LD_50_ of the water extract of *C. oxyacantha* leaves was measured as 13.5 g/kg, with no dead subjects and no behavioral changes from up to 10 g/kg [96]. The 70% ethanol extract of *C. aronia* leaves did not cause any behavioral changes, and there were no dead subjects up to 5000 mg/kg [97]. In addition, when the water extract of a whole plant of *C. aronia* (up to 2000 mg/kg, for 28 days) was administered orally to Wistar rats, there were no signs of acute toxicity or fatality [98]. Therefore, the LD_50_ of *C. aronia* leaves and the whole plant may possibly exceed 5000 mg/kg and 2000 mg/kg, respectively. Although the LD_50_ values vary depending on the hawthorn species, medicinal parts, and extraction methods, all the LD_50_ values in rodent models exceeded 2000 mg/kg, which is considered relatively safe compared to aspirin (LD_50_, 200 mg/kg) and metformin (LD_50_, 1000 mg/kg).

A systematic review demonstrated that the adverse events of *C*. extract WS^®^ 1442 (160–1800 mg/day for 3–24 weeks) include dizziness, gastrointestinal disturbance, headache, and palpitations [99], but another study emphasized that no significant adverse events and no specific drug interactions were observed after administration of WS^®^ 1442 [100].

As demonstrated above, hawthorn extracts displayed significant hepatoprotective activities against various stimulants, including fat diet, alcohol, heavy metals, and hepatectomy. Thus, considering animal studies and reviews of clinical trials on the safety of hawthorn, the plant can be regarded as a drug that can be prescribed with little toxicity in patients with liver diseases, despite further studies.

## 5. Discussion

Recently, herbal interventions have emerged as a therapeutic option for managing pathophysiological changes in various liver diseases, including NAFLD, viral hepatitis, and HCC [11,101]. Hawthorn is efficacious and has high potential to be developed to treat liver diseases. Despite the availability of increasing preclinical evidence of hawthorn, there is still ambiguity in confirming the pharmacological benefits and mechanisms of hawthorn extracts against liver-related pathological conditions. This review, therefore, includes significant advances in the pharmacological and molecular understanding of hawthorn extracts in liver disease management.

As demonstrated above, treatment with hawthorn extracts significantly reversed abnormalities related to hepatic injury, steatosis, inflammation, fibrosis, and cancer. These pathological conditions have been suggested to be closely and intricately linked with the risk of various liver diseases. Hence, hawthorn can be used as a therapeutic target for various hepatic diseases. In addition, the potential mechanisms covering antioxidant, apoptosis regulation, AMPK signaling, cholesterol metabolism, HSC inactivation, cell cycle arrest, and autophagy induction account for the pharmacological actions of hawthorn. Presumably, antioxidant defense by hawthorn extract against hepatic disorders is likely to be of interest, as many studies have determined the regulation of oxidative stress in hawthorn extract in reducing liver injury, fat accumulation, inflammation, and fibrosis. In terms of apoptosis, hawthorn extracts had a flexible impact in mediating apoptosis, in that hawthorn exerted hepatoprotective, anti-inflammatory, and antifibrotic activities through antiapoptosis in hepatic tissue, while the plant-induced apoptotic signal suppressed the cell viability of HepG2 cells. Moreover, the additional involvement of hawthorn in AMPK signaling, HSC inactivation, and hepatic cholesterol metabolism make the herb a possible agent for developing drugs for the management of liver diseases (Table 1, Table 2, Table 3 and Table 4, Figure 1, Figure 2, Figure 3 and Figure 4).

Collectively, liver diseases are a leading cause of worldwide morbidity and mortality. Recently, cases of HCC, ALD, NAFLD, and viral hepatitis have been increasing [102]. First, hawthorn possesses marked hepatoprotective, antisteatotic, anti-inflammatory, and antifibrotic effects against alcohol-induced liver injury, liver weight gain, fat deposition, hepatic inflammatory cell aggregation, and hepatic fibrosis. The beneficial properties of *C. pinnatifida* and *C. oxyacantha* against alcohol may be mainly implicated in ALDH and CYP2E1 enzyme-modulating activities and antioxidant effects, suggesting that these species can inhibit the overaccumulation of acetaldehyde, efficiently scavenge ROS attack, and reduce oxidation induced by alcohol.

Second, hawthorn treats NAFLD and metabolic syndromes, including diabetes, obesity, and hyperlipidemia. Supported by a large number of available preclinical results, hawthorn extract antagonized the significant changes in a variety of NAFLD rodent models. Specifically, hawthorn extracts were potent in reducing lipid content, inflammatory cell infiltration, and the expression of cytokines in liver tissue, thereby improving simple steatosis and steatohepatitis, mainly through antioxidant and AMPK signaling. The coordinated regulation of hawthorn extract on steatosis and inflammation may effectively prevent NASH-related fibrosis. Unfortunately, there was no preclinical evidence relating to the antifibrotic activity of hawthorn extracts in experimental models induced by Western diets, but the extract reduced liver fibrosis induced by CCl_4_ and alcohol treatment. Hence, this field merits further research. Nevertheless, hawthorn extracts might be an effective regulator of metabolic changes that occur during NAFLD because an increasing amount of experimental data support that pharmacological effects of the herb are antidiabetic, antiobetic, and hypolipidemic, resulting in low risks of metabolic syndrome and cardiovascular diseases. Hence, it is conceivable that hawthorn extracts, specifically haw pectin and polyphenols from *C. pinnatifida*, act as potent anti-NAFLD drugs that orchestrate complex metabolic changes occurring in NAFLD, thereby treating hepatic steatosis and inflammation.

Third, hawthorn extracts may be involved in the treatment of HCC, which is the most common and aggressive form of liver cancer, with high morbidity and mortality. Various curative therapies, including liver transplantation, surgical hepatectomy, and local ablation, are suitable for almost all early-stage cases. However, late diagnosis, inadequate hepatic reserves, and metastasis have lowered the therapeutic effects and 5-year survival rates of available treatment options [103]. Specifically, conventional chemotherapeutic agents often induce side effects by generating oxidative stress in non-targeted normal tissues. Similar to chemotherapy with cisplatin and 5-fluorouracil, hawthorn extracts also displayed anticancer effects against HepG2 cells. However, the abundance of antioxidant and hepatoprotective compounds constituting hawthorn extracts may reduce toxicity against normal cells. Although the effects of antioxidant and cytotoxic effects of hawthorn extract in HCC still need to be investigated, the triterpenoids, phenylpropanoids, and lignans obtained from *C. pinnatifida* might be crucial chemical groups for the control of HCC, as demonstrated above.

The present review also revealed the various species, parts, and compounds of hawthorn that were used to reduce hepatotoxicity, hepatic fat deposition, liver inflammation, fibrosis, and cancer in animal and cellular studies. The major species of hawthorn treated for alleviating hepatic pathological conditions are *C. pinnatifida*, *C. oxyacantha*, and *C. monogyna*. Among them, the fruits of *C. pinnatifida* can play a crucial role in improving hepatocytes overwhelmed by metabolic triggers leading to NAFLD by reducing oxidative stress and activating AMPK and NIK signaling. *C. oxyacantha* leaves and flowers may treat NASH-related fibrosis because the plant actively reduced hepatic fibrotic septa and collagen synthesis by CCl_4_ via HSC inactivation and antioxidant processes. *C. monogyna* buds and fruits may have selective cytotoxicity against HCC, with no harmful effects on normal cells because of their abundant antioxidant substances. The specific groups of active ingredients of hawthorn have been shown to be responsible for the following beneficial actions: (1) polyphenols obtained from hawthorn extracts displayed hepatoprotective and anti-inflammatory actions by regulating apoptosis and oxidative stress. (2) Haw pectin isolated from hawthorn fruits enhanced the clearance of hepatic accumulation of lipids through AMPK-dependent regulation, reduced hepatic oxidative stress and hepatic cholesterol catabolism with the regulation of hepatic inflammation via NFκB inactivation. (3) Triterpenoids, phenylpropanoids, and lignans of hawthorn plant were shown to have anticancer effects against HCC cell lines. In particular, vitexin, hyperoside, and corosolic acid were found in hawthorn to be among the major active ingredients primarily contributing to the bioactive activities of hawthorn against various liver diseases [104,105,106,107,108,109], and these compounds need further investigation to evaluate their efficacy.

In summary, the efficacy of hawthorn extract can be linked to ameliorating pathological conditions, including liver toxicity, steatosis, inflammation, fibrosis, and cancer of various hepatic disorders. The antioxidant mechanism of hawthorn extract may be crucial for the control of hepatic injury, steatosis, inflammation, and fibrosis. In addition to reducing oxidative stress in hepatic tissue by hawthorn extract, AMPK activation and antiapoptotic signaling also account for the improvement of hepatic steatosis and inflammation. Thus, given the role of hawthorn extracts in the regulation of hepatic disorders, the plant can contribute to the treatment of ALD, NAFLD, and HCC, as well as improve liver injury. However, further in vivo and clinical studies need to be elaborately designed and performed to confirm the clinical application of hawthorn extract and its compounds.

## 6. Conclusions

In conclusion, the present review demonstrates that hawthorn extracts have benefits against hepatic toxicity, fat deposition, inflammation, fibrosis, and cancer. The pharmacological activities of hawthorn extracts may be mainly due to reduced hepatic oxidative stress, which prevents excessive ROS attack and induces consequent hepatocellular function recovery. In addition, the regulation of AMPK, NIK, apoptosis, cholesterol metabolism, HSC activation, cell cycle arrest, and autophagy by hawthorn extracts is involved in its modulatory role in hepatic pathologic conditions. Therefore, hawthorn extracts may be promising and safe in treating hepatic disorders, even though in-depth and elaborate investigations on the efficacy, safety, and mechanism of action of the plant against liver diseases are needed.

## Figures and Tables

**Table 1:** Hepatoprotective effects and molecular mechanisms of hawthorn extract.

Sources	Models	Doses	Results and Mechanisms	Reference
Extract of *C. pinnatifida* (leaves)	In vivo, male Sprague Dawley rats fed with high-fat diets	100 mg/kg	↓**Liver damage induced by high-fat diet** ↓Serum ALP, LDH	[34]
Water extract of *C. pinnatifida* (fruits)	In vivo, male Sprague Dawley rats fed with high-fat diets	10 mL/kg	↓**Liver damage induced by high-fat diet** ↓Serum ALP, GGT ↓Liver pyknotic nuclei	[35]
Extract of hawthorn (leaves)	In vivo, male Sprague Dawley rats fed with high-fat diets	160 mg/kg	↓**Liver damage induced by high-fat diet** ↓Ballooning degeneration necrosis in liver tissue **Antioxidant** ↓Nrf2-positive stained hepatocytes ↑Hepatic Nrf2 mRNA ↓Hepatic GST, HO-1, rGCS mRNA and protein	[36]
Ethanolic extract of *C. oxycantha*	In vivo, male Wistar rats fed with high-fat diets	20 mg/kg	↓**Liver damage induced by high-fat diet** ↓Serum AST, ALT, GGT, ALP, total bilirubin, direct bilirubin, LDH, and MDA	[28]
70% ethanol extract of *C. aronia* (leaves and flowers)	In vivo, male Wistar rats fed with high-fat diets	200 mg/kg	↓**Liver damage induced by high-fat diet** ↓Highly vacuolated hepatocytes ↓Damaged endoplasmic reticuli ↓Distorted intercellular spaces ↓Irregular nuclear membranes **Antioxidant** ↑Hepatic GSH ↓Hepatic TBARS	[37]
Water extract of *C. aronia*	In vivo, male Wistar rats fed with high-fat diets	200 mg/kg	↓**Liver damage induced by high-fat diet** ↓Serum AST, ALT, and GGT	[29]
70% ethanol extract of *C. monogyna*	In vivo, male Wistar rats fed with high-cholesterol diet	100 mg/kg	↓**Liver damage induced by high cholesterol diet** ↓Serum AST, ALT, and GGT ↓Hepatic MDA protein ↓Highly vacuolated hepatocytes and nuclear chromatin condensation in liver tissue **Antioxidant** ↓Hepatic total thiol molecules ↑DPPH radical scavenging	[30]
70% ethanol extract of *C. pinnatifida*31 (leaves)	In vivo, Sprague Dawley rats fed with high-cholesterol diet	5, 7.5, 10 mL/kg	↓**Liver damage induced by high cholesterol diet** ↓Serum AST, ALT, and GGT ↓Cell necrosis, sinusoidal distension in liver tissue	[31]
80% ethanol extract of *C. pinnatifida* (fruits)	In vivo, male Sprague Dawley rats fed high-fat and high-cholesterol diets	5, 10%	↓**Liver damage induced by high fat and cholesterol diet** ↓Serum AST, ALT, ALP, and LDH	[32]
Powder from dried *C. pinnatifida* (leaves)	In vivo, Sprague Dawley rats fed with high-triglyceride diet	2%	↓**Liver damage induced by high triglyceride diet** ↓Serum ALT, ALP ↓Hepatocyte enlargement	[33]
Polyphenols from 80% ethanol extract of hawthorn peels and fleshes	In vivo, male Kunming mice fed with high-fructose diet	400 mg/kg	↓**Liver damage induced by high-fructose diet** ↓Serum AST, ALT, and ALP ↓Hepatic MDA protein ↓Hepatocyte necrosis, cytoplasmic vacuolation, cellular degeneration, and the loss of cellular boundaries in liver tissue **Antiapoptosis** ↓Hepatic Bax, Bax/Bcl-2 protein ↑Hepatic Bcl-2 protein (only in hawthorn peels) **Antioxidant** ↑Hepatic SOD, GSH-Px protein ↓Hepatic Nrf-2, ARE protein	[38]
Water extract of *C. pinnatifida* (fruits)	In vivo, male Sprague Dawley rats fed 25% alcohol for 55 days	1 cc/100 g	↓**Liver damage induced by alcohol** ↓Serum AST, ALT, ALP, and LDH	[39]
95% ethanol extract of *C. pinnatifida* (fruits)	In vivo, male Sprague Dawley rats fed 50% alcohol for 6 weeks	100 mg/kg	↓**Liver damage induced by alcohol** ↓Hepatic ADH activity ↑Hepatic ALDH activity	[40]
80% methanol extract of *C. pinnatifida*	In vitro, human hepatoma HepG2 cells induced by 1.3% ethanol	0.4%	↓**Liver damage induced by alcohol** ↑Cell viability 152.5% **Antioxidant** ↑DPPH radical scavenging ↓LDL oxidation	[10]
70% ethanol extract of dried *C. pinnatifida* (branches)	In vitro, human hepatoma HepG2 cells induced by 1.3% ethanol	0.4, 1%	↓**Liver damage induced by alcohol** ↑Cell viability 136.3% ↓Liver cell DNA damage ↓CYP2E1 enzyme expression ↓Catalytic activity of CYP2E1	[41]
Methanol extract of *C. oxycantha* (leaves)	In vivo, male Wistar rats administered with 3 g/kg/day of 35% ethanol	50 mg/kg	↓**Liver damage induced by alcohol** ↓Serum AST, ALT, GGT, ACP, and bilirubin ↑Liver glycogen ↓Hepatic MDA ↓Cell congestion, necrosis, and sinusoidal distension in liver tissue	[42]
70% ethanol extract of the leaves of *Crataegus pinnatifida* from a local market in China	In vivo, Sprague Dawley rats fed 56% alcohol for 8 weeks	5 mL/kg	↓**Liver damage induced by alcohol** ↓Serum AST, ALT, and GGT ↓Cell necrosis, sinusoidal distension in liver tissue	[31]
Flavonoids from *C. pinnatifida* (fruits)	In vivo, male Sprague Dawley rats injected with LPS	50, 100, 200 mg/kg	↓**Liver damage induced by LPS** ↓Serum AST, ALT ↓Extensive hepatocyte necrosis in liver tissue	[43]
Hawthorn capsule extracted from *C. oxyacantha* (leaves and flowers)	In vivo, male Wistar albino rats received an oral administration of CCl_4_	350 mg/kg	↓**Liver damage induced by toxic substances** ↓Serum AST, ALT, GGT, and bilirubin ↑Serum albumin ↓Hepatic MDA, **Antioxidant** ↓Hepatic MPO activity ↓Hepatic P. Carbonyl activity ↑Hepatic SOD activity	[44]
Extract of hawthorn	In vivo, Sprague Dawley rats of both sexes received an oral administration of CCl_4_	40 mg/kg	↓**Liver damage induced by toxic substances** ↓Serum AST, ALT, and ALP ↓Vacuolar degeneration in hepatocytes ↑Protein and mucopolysaccharide contents in hepatocytes	[45]
Water extract of *C. pinnatifida* (fruits)	In vitro, Rat hepatocytes H4IIE induced by cadmium	0.1, 0.3 mg/mL	↓**Liver damage induced by heavy metals** ↑Cell viability **Antiapoptosis** ↓PARP cleavage	[46]
	In vivo, male Sprague Dawley rats intravenously injected with cadmium 4 mg/kg	50, 100 mg/kg	↓**Liver damage induced by heavy metals** ↓Serum AST, ALT, and LDH Hepatic degenerative regions and cells Hepatic centrolobular necrosis with peripheral hemorrhages/congestions	
70% ethyl alcohol extract of *C. aronia* (flowers)	In vivo, male albino rats with 50% partial hepatectomy	0.5, 1%	↓**Liver damage induced by partial hepatectomy** ↓Serum AST, ALT **Antiapoptosis** ↓TUNEL-positive hepatocytes	[47]

ACP, acid phosphatase; ADH, alcohol dehydrogenase; ALDH, aldehyde dehydrogenase; ALP, alkaline phosphatase; ALT, alanine transaminase; ARE, antioxidant response element; AST, aspartate transaminase; Bax, Bcl-2-associated X protein; Bcl-2, B-cell lymphoma-2; CCl_4_, carbon tetrachloride; CYP2E1, cytochrome P450 2E1; DPPH, 2,2-diphenyl-1-picrylhydrazyl; GGT, gamma glutamyl transpeptidase; GSH, glutathione; GSH-Px, glutathione peroxidase; GST, glutathione S-transferase; HO-1, heme oxygenase-1; LDH, lactate dehydrogenase; LDL, low-density lipoprotein; LPS, lipopolysaccharides; MDA, malondialdehyde; MPO, myeloperoxidase; Nrf-2, nuclear factor erythroid-2-related factor 2; PARP, poly-ADP ribose polymerase; P. Carbonyl, protein carbonyl; rGCS, r-glutamylcysteine synthethase; SOD, superoxide dismutase; TBARS, thiobarbituric acid reactive substances; and TUNEL, terminal deoxynucleotidyl transferase dUTP nick end labelling.

**Table 2:** Antisteatotic effects and molecular mechanisms of hawthorn extract.

Sources	Models	Doses	Results and Mechanisms	Reference
Extract of hawthorn (leaves)	In vivo, male Sprague Dawley rats fed with high-fat diets	160 mg/kg	↓**Hepatic steatosis induced by high-fat diet** ↓Fat deposition in liver tissue **Antioxidant** ↑Nrf2-positive stained hepatocytes ↑Hepatic Nrf2 mRNA ↓Hepatic GST, HO-1, rGCS mRNA, and protein	[36]
Extract of *C. pinnatifida* (leaves)	In vivo, male Sprague Dawley rats fed with high-fat diets	100 mg/kg	↓**Hepatic steatosis induced by high-fat diet** ↓Liver weight ↓Fat deposition in liver tissue ↓Hepatic TC, TG **Adiponectin/AMPK signaling** ↑Serum adiponectin ↑Hepatic adiponectin receptor 2 mRNA and protein ↑Hepatic p-AMPKα protein ↓Hepatic SREBP-1c mRNA and protein ↑Hepatic PPARα mRNA and protein ↓Heptatic CD36, FAS, and SCD1 mRNA ↑Hepatic CPT1, ACO, and ACOX1 mRNA	[34]
Haw pectin pentaoligosaccharide from *C. pinnatifida* (fruits)	In vivo, male Kunming mice fed with high-fat diets	150 mg/kg	↓**Hepatic steatosis induced by high-fat diet** ↓Fat deposition in liver tissue **AMPK signaling** ↑Hepatic CPT1, ACO mRNA ↑Hepatic PPARα mRNA and protein	[53]
Haw pectin from *C. pinnatifida* (fruits)	In vivo, male Kunming mice fed with high-fat diets	50, 150, 300 mg/kg	↓**Hepatic steatosis induced by high-fat diet** ↓Hepatic TC **Hepatic cholesterol and bile acid metabolism** ↓Hepatic HMG-CoA reductase, ACAT mRNA, and protein ↑Hepatic cholesterol 7α-hydroxylase mRNA and protein	[54]
Haw pectin from *C. pinnatifida* (fruits)	In vivo, male Kunming mice fed with high-fat diets	300 mg/kg	↓**Hepatic steatosis induced by high-fat diet** ↓Liver weight ↓Hepatic TC **Hepatic cholesterol and bile acid metabolism** ↓Hepatic bile acids ↑Gallbladder bile acids ↑Hepatic cholesterol 7α-hydroxylase mRNA and protein ↑Hepatic ABCA1, SR-BI, LXRα, BSEP mRNA, and protein	[55]
Haw pectin from *C. pinnatifida* (fruits)	In vivo, male Kunming mice fed with high-fat diets	300 mg/kg	↓**Hepatic steatosis induced by high-fat diet** ↓Hepatic TC **Hepatic cholesterol and bile acid metabolism** ↓Hepatic FGFR4 mRNA and protein ↑Hepatic cholesterol 7α-hydroxylase mRNA and protein ↑Fecal bile acids ↓Hepatic bile acids	[56]
Haw pectin from *C. pinnatifida* (fruits)	In vivo, male Kunming mice fed with high-fat diets	50, 150, 300 mg/kg	↓**Hepatic steatosis induced by high-fat diet** ↓Liver weight ↓Fat deposition in liver tissue **Antioxidant** ↑Hepatic SOD activity	[74]
Haw pectin from *C. pinnatifida* (fruits)	In vivo, male Kunming mice fed with high-fat diets	50, 150, 300 mg/kg	↓**Hepatic steatosis induced by high-fat diet** ↓Hepatic TG **Antioxidant** ↑Hepatic SOD, CAT, and GSH-Px activity ↑Hepatic TAC, GSH levels	[75]
Haw pectin from *C. pinnatifida* (fruits)	In vivo, male Kunming mice fed with high-fat diets	150 mg/kg	↓**Hepatic steatosis induced by high-fat diet** ↓Liver weight ↓Fat deposition in liver tissue ↓Hepatic TG, total lipids **AMPK/SIRT1/NFκB signaling** ↑Hepatic AMPKα, SIRT1 mRNA ↓Hepatic NFκB mRNA and protein	[77]
70% ethanol extract of *C. aronia* (leaves and flowers)	In vivo, male Wistar rats fed with high-fat diets	200 mg/kg	↓**Hepatic steatosis induced by high-fat diet** ↓Fat deposition in liver tissue **Antioxidant** ↑Hepatic GSH ↓Hepatic TBARS	[37]
80% ethanol extract of *C. pinnatifida* (fruits)	In vivo, male Sprague Dawley rats fed high-cholesterol diet	2%	↓**Hepatic steatosis induced by high-cholesterol diet** ↓Liver weight ↓Fat deposition in liver tissue **Antioxidant** ↑Hepatic SOD, CAT activity	[57]
70% ethanol extract of *C. monogyna*	In vivo, male Wistar rats fed with high-cholesterol diet	100 mg/kg	↓**Hepatic steatosis induced by high-cholesterol diet** ↓Liver/body weight ↓Hepatic TC, TG, and LDL **Antioxidant** ↓Hepatic total thiol molecules ↑DPPH radical scavenging	[30]
Water extract of *C. pinnatifida* (fruits)	In vivo, male Sprague Dawley rats fed with high-fat diets	397.3 mg/kg	↓**Hepatic steatosis induced by high-cholesterol diet** ↓Liver weight	[58]
80% ethanol extract of *C. pinnatifida* (fruits)	In vivo, male Sprague Dawley rats fed with high-fat and high-cholesterol diets	5, 10%	↓**Hepatic steatosis induced by high-fat and cholesterol diet** ↓Fat deposition in liver tissue ↓Hepatic TC, TG	[32]
80% ethanol extract of *C. pinnatifida* (fruits)	In vivo, male Sprague Dawley rats fed with high-cholesterol diet	100 mg/kg	↓**Hepatic steatosis induced by high-cholesterol diet** ↓Hepatic lipid contents **Hepatic cholesterol metabolism** ↑Hepatic cholesterol 7α-hydroxylase mRNA	[59]
80% ethanol extract of *C. pinnatifida* (fruits)	In vivo, male Syrian golden hamsters fed with high-cholesterol diet	0.5%	↓**Hepatic steatosis induced by high-cholesterol diet** ↓Hepatic FFA contents **Hepatic cholesterol metabolism** ↑Hepatic cholesterol 7α-hydroxylase protein	[60]
Water extract of *C. pinnatifida* (fruits)	In vivo, male Sprague Dawley rats fed with high-cholesterol diet	2%	↓**Hepatic steatosis induced by high-cholesterol diet** ↓Hepatic TC, TG **Hepatic cholesterol metabolism** ↓Hepatic ACAT activity	[61]
Water extract of *C. pinnatifida* (fruits)	In vivo, male Sprague Dawley rats fed with high-cholesterol diet	124 mg/kg	↓**Hepatic steatosis induced by high-cholesterol diet** ↓Hepatic lipid contents	[62]
Water extract of *C. pinnatifida* (fruits)	In vivo, female ICR mice fed with high-cholesterol diet	50, 100 mg/kg	↓**Hepatic steatosis induced by high-cholesterol diet** ↓Liver weight ↓Hepatic TC, TG	[63]
70% ethanol extract of *C. pinnatifida* (fruits)	In vivo, male Sprague Dawley rats fed with high-fat diets	500, 1000 mg/kg	↓**Hepatic steatosis induced by high-fat diet** ↓Hepatic TC, TG	[64]
Water extract of *C. pinnatifida* (fruits)	In vivo, male Sprague Dawley rats fed with high-fat diets	10 mL/kg	↓**Hepatic steatosis induced by high-fat diet** ↓Liver weight ↓Fat deposition in liver tissue ↓Hepatic TC, TG	[65]
Water extract of *C. pinnatifida* (fruits)	In vivo, male Sprague Dawley rats fed with high-fat diets	10 mL/kg	↓**Hepatic steatosis induced by high-fat diet** ↓Fat deposition in liver tissue	[35]
Methanol extract of *C. pinnatifida* (fruits)	In vivo, female ICR mice fed with high-fat diets	100 μg	↓**Hepatic steatosis induced by high-fat diet** ↓Liver weight	[66]
95% ethanol extract of *C. cuneata* (fruits)	In vivo, male Kunming mice fed with high-fat diet	90, 130 mg/kg	↓**Hepatic steatosis induced by high-cholesterol diet** ↓Fat deposition in liver tissue	[67]
Ethanol extract of *C. cuneata* (fruits)	In vivo, male mice fed with high-fat diet	130 mg/kg	↓**Hepatic steatosis induced by high-fat diet** ↓Hepatic TC **Hepatic cholesterol metabolism** ↓Hepatic HMG-CoA reductase mRNA ↓Hepatic HMG-CoA reductase promoter activity ↓Hepatic NFκB p65 mRNA	[68]
Water extract of *C. aronia* (herba)	In vivo, male Wistar rats fed with high-fat diet	200 mg/kg	↓**Hepatic steatosis induced by high-fat diet** ↓Liver weight ↓Fat deposition in liver tissue	[29]
70% ethanol extract of *C. pinnatifida* (leaves)	In vivo, Sprague Dawley rats fed with high-cholesterol diet	5, 7.5, 10 mL/kg	↓**Hepatic steatosis induced by high-cholesterol diet** ↓Liver weight ↓Fat deposition in liver tissue	[31]
Ethanol extract of *C. oxycantha*	In vivo, male Wistar rats fed high-fat diet	20 mg/kg	↓**Hepatic steatosis induced by high-fat diet** ↓Fat deposition in liver tissue	[28]
Water extract of *C. aronia* (fruits)	In vivo, New Zealand white rabbits fed high-fat diet	10 mg/kg	↓**Hepatic steatosis induced by high-fat diet** ↓Hepatic TC, TG, FFA, and phospholipids **Hepatic cholesterol metabolism** ↓Hepatic HMG-CoA reductase, ACAT activity ↑Hepatic cholesterol 7-hydroxylase activity	[69]
Polyphenols from 80% ethanol extracs of hawthorn peels and fleshes	In vivo, male Kunming mice fed with high-fructose diet	400 mg/kg	↓**Hepatic steatosis induced by high-fructose diet** ↓Liver weight ↓Fat deposition in liver tissue ↓Hepatic TC, TG **Antioxidant** ↑Hepatic SOD, GSH-Px protein ↓Hepatic Nrf-2, ARE protein **AMPK signaling** ↑Hepatic PPARα protein ↓Hepatic FAS protein	[38]
Ethanol extract of *C. oxycantha* (fruits)	In vivo, male Wistar rats fed atherogenic diet	0.5 mL/100 g	↓**Hepatic steatosis induced by atherogenic diet** ↓Hepatic TC **Hepatic cholesterol catabolism to bile acids** ↑Hepatic LDL receptors ↓Hepatic cholesterol biosynthesis ↑Hepatic bile acids ↑Fecal bile acidss	[70]
80% ethanol extract of hawthorn (fruits)	In vivo, ApoE^−/−^ mice fed atherogenic diet	2%	↓**Hepatic steatosis induced by atherogenic diet** ↓Hepatic TC ↓Hepatic fatty acids **Antioxidant** ↑Hepatic TAC protein ↑Hepatic SOD mRNA and protein ↑Hepatic GSH-Px mRNA ↑Hepatic CAT mRNA and protein	[71]
70% ethanol extract of *C. pinnatifida* (fruits)	In vivo, male C57BL/6J mice fed MCD diet	300 mg/kg	↓**Hepatic steatosis induced by MCD diet** ↓Fat deposition in liver tissue ↓Hepatic TG **AMPK signaling** ↑Hepatic p-AMPKα protein ↓Hepatic SREBP-1c, C/EBPα, and PPARγ protein ↓Hepatic ACC, FAS protein	[72]
70% ethanol extract of *C. pinnatifida* (fruits)	In vivo, female ovariectomized Sprague Dawley rats	100, 200 mg/kg	↓**Hepatic steatosis after ovariectomy** ↓Hepatic TG **Antioxidant** ↑Hepatic Nrf2 mRNA and protein ↑Hepatic HO-1 mRNA and protein ↑Hepatic GSH-Px mRNA and protein ↑Hepatic CAT mRNA and protein	[73]
Water extract of *C. pinnatifida* (fruits)	In vivo, male Sprague Dawley rats fed 25% alcohol for 55 days	1 cc/100 g	↓**Hepatic steatosis induced by alcohol** ↓Liver weight	[39]
70% ethanol extract of *C. pinnatifida* (leaves)	In vivo, Sprague Dawley rats fed 56% alcohol for 8 weeks	5 mL/kg	↓**Hepatic steatosis induced by alcohol** ↓Liver weight ↓Fat deposition in liver tissue	[31]

ABCA1, ABC transporters A1; ACAT, acyl-CoA: cholesterol acyltransferase; ACC, acetyl-CoA carboxylase; ACO, acyl-CoA oxidase; ACOX1, acyl-CoA oxidase 1; AMPK, AMP-activated protein kinase; ARE, antioxidant response element; BSEP, bile salt export pump; CAT, catalase; C/EBP, CCAAT-enhancer-binding proteins; CPT1, carnitine palmitoyltransferase-1; FAS, fatty acid synthase; FFA, free fatty acids; FGFR4, fibroblast growth factor receptor 4; GSH, glutathione; GSH-Px, glutathione peroxidase; GST, glutathione S-transferase; HO-1, heme oxygenase-1; LXR, liver X receptor; MCD, methionine choline deficient; NFκB, nuclear factor kappa B; Nrf2, nuclear factor erythroid-2-related factor 2; PPAR, peroxisome proliferator-activated receptor; rGCS, r-glutamylcysteine synthethase; SCD1, stearoyl CoA desaturase 1; SIRT, silent information regulator T; SOD, superoxide dismutase; SR-BI, scavenger receptor class B type I; SREBP, sterol regulatory element binding protein; TAC, total antioxidant capacity; TBARS, thiobarbituric acid reactive substances; TC, total cholesterol; and TG, triglyceride.

**Table 3:** Anti-inflammatory and antifibrotic effects and molecular mechanisms of hawthorn extract.

Sources	Models	Doses	Results and Mechanisms	Reference
Hawthorn capsule extracted from *C. oxyacantha* (leaves and flowers)	In vivo, male Wistar albino rats orally administered with CCl_4_	350 mg/kg	↓**Hepatic inflammation induced by** **CCl_4_** ↓Hepatic IL-1β, TNF-α mRNA ↓Hepatic COX-2 mRNA **Antioxidant** ↓Hepatic MPO activity ↓Hepatic P. carbonyl contents ↑Hepatic SOD activity **NFκB signaling** ↓Hepatic NFκB mRNA	[44]
Flavonoids from *C. pinnatifida* (fruits)	In vivo, male Sprague Dawley rats injected with LPS	50, 100, 200 mg/kg	↓**Hepatic inflammation induced by LPS** ↓Neutrophil leukocyte infiltration in liver tissue ↓Hepatic expression of iNOS and COX-2	[43]
80% ethanol extract of *C. pinnatifida* (fruits)	In vivo, male Sprague Dawley rats fed with high-cholesterol diets	2%	↓**Hepatic inflammation induced by high-cholesterol diet** ↓Hepatic expression of NOS **Antioxidant** ↑Hepatic SOD, CAT activity	[57]
70% ethanol extract of *C. monogyna*	In vivo, male Wistar rats fed with high-cholesterol diets	100 mg/kg	↓**Hepatic inflammation induced by high-cholesterol diet** ↓Inflammatory cells infiltration in liver tissue **Antioxidant** ↓Hepatic total thiol molecules ↑DPPH radical scavenging	[30]
70% ethanol extract of *C. pinnatifida* (leaves)	In vivo, Sprague Dawley rats fed with high-cholesterol diets	5, 7.5, 10 mL/kg	↓**Hepatic inflammation induced by high cholesterol diet** ↓Inflammatory cells infiltration in liver tissue	[31]
Powder from dried *C. pinnatifida* (leaves)	In vivo, Sprague Dawley rats fed with high-triglyceride diets	2%	↓**Hepatic inflammation induced by high triglyceride diet** ↓Mononuclear inflammatory cells around the hepatic blood vessels	[33]
Haw pectin from the water extract of *C. pinnatifida*	In vivo, male Kunming mice fed with high-fat diets	150 mg/kg	↓**Hepatic inflammation induced by high-fat diet** ↓Hepatic TNF-α, IL-6 contents ↑Hepatic IL-10 contents **NIK/IKK/NFκB signaling** ↓Hepatic RIP1, NIK, IKKα, TNFα, TNFR1, and TRAF2 mRNA ↓Hepatic NFκB mRNA and protein **AMPK/SIRT1/NFκB signaling** ↑Hepatic AMPKα, SIRT1 mRNA ↓Hepatic NFκB mRNA and protein	[77]
Polyphenols from the ethanol extract of *C. pinnatifida* (fruits)	In vivo, male Wistar rats fed with high-fat diets and streptozotocin	300 mg/kg	↓**Hepatic inflammation induced by high-fat diet and streptozotocin** ↓Inflammatory cells infiltration in liver tissue ↓Hepatic TNF-α, MCP-1, and IL-6 protein **AMPK/SIRT1/NFκB signaling** ↑Hepatic AMPKα, PPARδ, and SIRT1 protein ↓NFκB p65 protein	[82]
Water extract of hawthorn fruits and leaves	In vivo, male Sprague Dawley rats fed with high-fat diets	1 mg/100 g	↓**Hepatic inflammation induced by high-fat diet** ↓Inflammatory cells infiltration in liver tissue	[35]
80% ethanol extract of hawthorn (fruits)	In vivo, ApoE^−/−^ mice fed with atherogenic diets	2%	↓**Hepatic inflammation induced by atherogenic diet** ↓Hepatic MCP-1, TNF-α, IL-1β, IL-6, and IL-10 mRNA and protein **Antioxidant** ↑Hepatic TAC protein ↑Hepatic SOD mRNA and protein ↑Hepatic GSH-Px mRNA ↑Hepatic CAT mRNA and protein	[71]
Polyphenols from 80% ethanol extract of hawthorn peels and fleshes	In vivo, male Kunming mice fed with high-fructose diets	400 mg/kg	↓**Hepatic inflammation induced by high-fructose diet** ↓Hepatic expression of IL-1, IL-6, and TNF-α **Antiapoptosis** ↓Hepatic Bax, Bax/Bcl-2 protein ↑Hepatic Bcl-2 protein (only in hawthorn peels) **Antioxidant** ↓Hepatic MDA protein ↑Hepatic SOD, GSH-Px protein ↓Hepatic Nrf-2, ARE protein **AMPK signaling** ↑Hepatic PPARα protein	[38]
70% ethanol extract of *C. pinnatifida* (leaves)	In vivo, Sprague Dawley rats fed with 56% alcohol for 8 weeks	5 mL/kg	↓**Hepatic inflammation induced by alcohol** ↓Inflammatory cells infiltration in liver tissue	[31]
Extract of hawthorn	In vivo, Sprague Dawley rats orally administered with CCl_4_	40 mg/kg	↓**Liver fibrosis induced by toxic substances** ↓Marked fibrosis around the main blood vessels in liver tissue	[45]
Hawthorn capsule extracted from *C. oxyacantha* (leaves and flowers)	In vivo, male Wistar albino rats orally administered with CCl_4_	350 mg/kg	↓**Liver fibrosis induced by toxic substances** ↓Hepatic fibrotic septa ↓Severity score of Masson ↓Masson-positive area ↓Hepatic hydroxyproline protein ↓Hepatic collagen 1 and 3 protein and mRNA **HSC inactivation** ↓Hepatic α-SMA-positive cells ↓Hepatic α-SMA mRNA ↓Hepatic TGF-β mRNA **Antioxidant** ↓Liver MPO activity ↓Liver P. carbonyl contents ↑Liver SOD activity	[44]
Methanol extract of *C. oxycantha* (leaves)	In vivo, male Wistar rats administered with 3 g/kg/day of 35% ethanol	50 mg/kg	↓**Liver fibrosis induced by alcohol** ↓Moderate fibrosis near lobule central veins in liver tissue **Antioxidant** ↓Hepatic MDA	[42]

AMPK, AMP-activated protein kinase; ARE, antioxidant response element; Bax, Bcl-2-associated X protein; Bcl-2, B-cell lymphoma-2; CAT, catalase; CCl_4_, carbon tetrachloride; COX, Cyclooxygenase; DPPH, 2,2-diphenyl-1-picrylhydrazyl; GSH-Px, glutathione peroxidase; HSC, hepatic stellate cell; IKK, IκB kinase; IL, interleukin; iNOS, inducible nitric oxide synthase; LPS, lipopolysaccharides; MCP, monocyte chemoattractant protein; MDA, malondialdehyde; MPO, myeloperoxidase; NFκB, nuclear factor kappa B; NIK, NFκB inducing kinase; NOS, nitric oxide synthase; Nrf-2, nuclear factor erythroid-2-related factor 2; P. carbonyl, protein carbonyl; PPAR, peroxisome proliferator-activated receptor; RIP, receptor interacting protein kinase; SIRT, silent information regulator T; SOD, superoxide dismutase; SMA, smooth muscle actin; TAC, total antioxidant capacity; TGF, transforming growth factor; TNF, tumor necrosis factor; TNFR, TNF receptor; and TRAF, TNF receptor-associated factor.

**Table 4:** Anticancer effects and molecular mechanisms of hawthorn extract and its compounds.

Sources	Models	Doses	Results and Mechanisms	Reference
Ethanol extract of *C. pinnatifida* (fruits)	In vitro, human hepatoma HepG2 cells	0.8 mg/mL	↓**Cell viability** **Apoptosis induction** ↑Caspase-3 mRNA ↓Bcl-2 mRNA and protein ↑Bax mRNA and protein	[86]
80% ethanol extract of *C. monogyna* (buds and fruits)	In vitro, human hepatoma HepG2 cells	0.5, 1 mg/mL	↓**Cell viability**	[87]
Triterpenoids from 80% acetone extract of *C. pinnatifida* (fruits)	In vitro, human hepatoma HepG2 cells		↓**Cell viability** (IC_50_ < 5 μM)	[88]
Phenylpropanoids from 70% ethanol extract of *C. pinnatifida* (fruits)	In vitro, human hepatoma HepG2 and Hep3B cells	100 μM.	↓**Cell viability** (IC_50_: 17.5–27.36 μM in HepG2 and 38.96–43.58 μM in Hep3B) **Apoptosis induction**	[89]
Phenylpropanoids from 70% ethanol extract of *C. pinnatifida* (fruits)	In vitro, human hepatoma HepG2 and Hep3B cells	50 μM	↓**Cell viability** **Apoptosis induction** ↑Apoptotic cells **Autophagy induction** Monodansylcadaverine positive cells **Cell cycle arrest** **G2/M arrest by** (−)-crataegusanoid A **G0/G1 arrest by** (−)-crataegusanoid B	[94]
Lignans from 70% ethanol extract of *C. pinnatifida* (seeds)	In vitro, human hepatoma HepG2 cells		↓**Cell viability** (IC_50_: 38.54–39.97 μM)	[92]
80% methanol extract of *C. monogyna* (buds and fruits)	In vitro, human hepatoma HepG2 cells		↓**Cell viability**	[91]
80% acetone extract of *C. pinnatifida* obtained from Shandong Institue of (fruits)	In vitro, human hepatoma HepG2 cells	5–30 μg/ml	↓**Cell viability** (IC_50_: 11.58 μg/mL) ↓**Cell proliferation** IC_50_ of ursolic acid 12.58 μM IC_50_ of corosolic acid 9.44 μM IC_50_ of maslinic acid 23.42 μM IC_50_ of oleanolic acid 54.02 μM	[20]
Dihydrobenzofuran neolignan from 70% ethanol extract of *C. pinnatifida* (seeds)	In vitro, human hepatoma HepG2 cells		↓**Cell viability** (IC_50_ = 30.96 μM)	[93]
80% ethanol extract of of *C. armena* (shoots, flowers, and fruits)	In vitro, human hepatoma HepG2 cells		↓**Cell viability** (IC_50_ = 8.66 μg/mL)	[90]

HepG2, hepatoma G2; Hep3B, hepatoma 3B; Bax, Bcl-2-associated X protein; Bcl-2, B-cell lymphoma-2; and IC_50_, half-maximal inhibitory concentration.

## Data Availability

Not applicable.
